# Necrotic Enteritis in Broiler Chickens: The Role of Tight Junctions and Mucosal Immune Responses in Alleviating the Effect of the Disease

**DOI:** 10.3390/microorganisms7080231

**Published:** 2019-07-31

**Authors:** Nima K. Emami, Ali Calik, Mallory B. White, Mark Young, Rami A. Dalloul

**Affiliations:** 1 Avian Immunobiology Laboratory, Department of Animal and Poultry Sciences, Virginia Tech, Blacksburg, VA 24061, USA; 2 Department of Animal Nutrition and Nutritional Diseases, Faculty of Veterinary Medicine, Ankara University, 06110 Ankara, Turkey; 3 Star-Labs/Forage Research, Inc., Clarksdale, MO 64430, USA

**Keywords:** necrotic enteritis, performance, lesion score, tight junction, immune response

## Abstract

Necrotic enteritis (NE) continues to present major challenges to the poultry industry, and the etiologic agent *Clostridium perfringens* is the fourth leading cause of bacterially-induced food- borne illnesses in the US. This study was designed to evaluate the effects of a probiotic during naturally occurring NE. On day of hatch, 1080 Cobb 500 male broilers were randomly allocated to three groups (12 replicate pens/treatment, 30 birds/pen) including (1) negative control (NC): corn-soybean meal diet; (2) positive control (PC): NC + 20 mg virginiamycin/kg diet (0.450 kg Stafac^®^20/ton); and (3) NC + PrimaLac (1.36 and 0.91 kg/ton from 1–21 and 22–42 days, respectively). One day (d) post placement, all birds were challenged by a commercial live oocyst coccidia vaccine as a predisposing factor to NE. Body weight and feed intake were measured at the onset of NE (d 8) and end of each feeding phase. On d 8, small intestines of two birds/pen were examined for NE lesions, and jejunum samples from one bird were collected for mRNA gene expression analysis of tight junction proteins, cytokines, and nutrient transporters. Data were analyzed using the JMP software and significance between treatments identified by LSD (*p* < 0.05). Compared to NC, supplementation of probiotic reduced d 1–42 mortality; however, PC was the only group with significantly lower mortality. Despite significantly improved feed conversion ratio (FCR) in PC and probiotic groups during d 1–42, average daily gain was only higher in PC (77.69 g/bird) compared with NC (74.99 g/bird). Furthermore, probiotic and PC groups had significantly reduced lesion scores in the duodenum and jejunum compared to NC. Expression of claudin-3 was higher, while expression of zonula occluden-2 tended (*p* = 0.06) to be higher in probiotic-supplemented birds compared to NC. Moreover, birds fed the probiotic diet had significantly higher expression of IL-10, IL-17, AMPK-α1, and SGLT1 mRNA compared to NC birds. The expression of PepT1 was higher for the probiotic-supplemented group compared to PC. IFN-γ expression was lower in PC compared to NC, while there was no difference between probiotic and NC. There were no differences in gene expression of sIgA, TNF-α, IL-1β, and IL-22 among treatments. Collectively, these data indicate that in a naturally occurring NE model, supplementation of a probiotic helps to improve FCR and reduce lesions, potentially due to the improvements in mRNA expression of tight junctions, cytokines, and nutrient transporters.

## 1. Introduction

Necrotic enteritis (NE) is a significant enteric disease in poultry with considerable economic effect on profitability. The annual financial loss to the poultry industry worldwide is estimated at up to $6 billion [1]. Additionally, *Clostridium perfringens* is the fourth leading cause of bacterially-induced food-borne illnesses in the US with $342 million loss/year [2]. To date, several nutraceutical supplements and vaccines have been used to reduce the incidence of necrotic enteritis [3]; however, despite years of research, necrotic enteritis is still one of the major challenges to the poultry industry [4]. *C*. *perfringens* is ubiquitous (found in soil, sewage, litter, feces, normal intestinal microbiota, foods) and poultry are constantly exposed to and ingest *C*. *perfringens* type A or their spores [5,6,7]. In healthy birds, *C*. *perfringens* population is ~10^2^–10^4^ CFU/g digesta; however, at the time of disease this number rises to 10^7^–10^9^ CFU/g digesta [8]. Therefore, it is now accepted that NE is a complex enteric disease that is caused by *C*. *perfringens*, but additional predisposing factors should be present to make the gut environment suitable for these bacteria to replicate and produce toxins [5,9]. Predisposing factors such as *Eimeria* parasites would lead to disturbances in the microbiota by disrupting the gastrointestinal environment, therefore allowing the proliferation of *C*. *perfringens* by either providing favorable ecological circumstances or nutrients [8,10]. In addition, damage to the epithelium caused by *Eimeria* leads to the release of serum and other nutrients from host cells or causes mucogenesis, which helps aid the proliferation of *Clostridium* sp. [11] and leads to changes in the gastrointestinal tract microbial profile [12].

An intact intestinal epithelium serves as a vital barrier preventing entry of potential pathogens and results in proper nutrient absorption and utilization, leading to optimal health and performance of the bird [13]. Tight junctions, which seal the paracellular space between adjacent epithelial cells, are required for the maintenance of the mucosal barrier [14]. *C*. *perfringens* enterotoxins (CPE) bind to tight junction proteins, mainly claudin-3 and claudin-4 [15,16], which eventually leads to pore formation, an increase in paracellular permeability, and cytotoxicity [16,17]. Therefore, during NE, tight junction structure would be compromised, thus influencing barrier function and eventually leading to lower performance and higher mortality in birds.

The intestine has a high requirement for energy to maintain its integrity and function, thus alteration in the intestinal energy metabolism and mitochondrial functionality may increase intestinal damage during an enteric disease challenge [18]. Adenosine monophosphate-activated protein kinase (AMPK), a sensor of cellular energy status, has a regulatory function on intestinal barrier function and epithelial differentiation, inflammatory response, and nutrient uptake [19,20,21]. All these complex pathways are closely associated with each other and changes in their regulation inevitably influence the health and performance status of broiler chickens. Pathogen invasion and penetration into the enterocytes triggers a cascade of signaling events in the intestine leading to secretion of various cytokines, which subsequently influence the integrity and function of the intestinal barrier, nutrient uptake, and epithelial cell energy metabolism. Based on findings that suggest the benefits of probiotic administration during enteric disease challenges, the current study hypothesized that dietary probiotic supplementation may alleviate the NE-induced growth reduction and pathology by regulating the expression of cytokines, tight junction proteins, and nutrient transporters. 

## 2. Materials and Methods

### 2.1. Birds, Housing, and Diets

A total of 1080 day-old Cobb 500 male broiler chickens were acquired from a local hatchery. Prior to placement, birds were weighed in groups of 30 each and allocated to one of 36 pens (~1.2 × 2.4 m; 32 sq. ft.). Birds were assigned to one of the three dietary treatments (12 pens/treatment) as follows: Negative Control (NC): corn-soybean meal basal diet (Table 1); Positive control (PC): NC + 20 mg virginiamycin/kg (as 0.450 kg Stafac^®^20) diet from d 0–42; Probiotic: NC + PrimaLac (1.36 kg/ton in the starter and grower 1 diets; 0.91 kg/ton in grower 2 and finisher diets). PrimaLac contains *Lactobacillus acidophilus*, *Lactobacillus casei*, *Bifidobacterium bifidum*, and *Enterococcus faecium* [22]. All animal protocols were approved and conducted under the guidelines of the Virginia Tech Institutional Animal Care and Use Committee (IACUC # 18-011).

One large batch of the basal diet was mixed and then divided equally for the three dietary treatments. Each of the additives was pre-mixed in 50 kg basal feed and then blended with the rest of the basal diet to reach the required amount of feed needed per treatment. The mash diets were subsequently crumbled (starter phase) or pelleted (grower and finisher phases). 

Each pen was equipped with a bucket-type feeder and a nipple drinker line with fresh wood shavings as litter (6 cm). Birds had ad libitum access to water and feed from placement (d 0) until the end of the study (d 42). Lighting schedule was 24 h light for the first three days, reduced to 23 h light: 1 h dark from d 4–7, and reduced further to 18 h light and 6 h dark thereafter. An automatic ventilation system was used to control the environment, and temperature was maintained as follows: 32 °C for the first 3 days, then gradually reduced approximately 3 °C each week until it reached 23 °C at the start of week 4 where it remained constant thereafter.

### 2.2. Necrotic Enteritis Challenge

In order to simulate field conditions, a unique, naturally occurring model developed on our research farm was applied to induce NE. This model consists of spraying a concentrated commercial coccidiosis vaccine on litter and feed upon bird placement, which, in conjunction with the presence of *C*. *perfringens* spores in the barn environment, leads to the development of a NE outbreak one week post vaccine application. For this trial, the Coccivac^®^-B52 vaccine (containing live oocysts of *Eimeria acervulina*, *E*. *maxima*, *E*. *maxima* MFP, *E*. *mivati*, and *E*. *tenella*; Merck Animal Health) was prepared at the proper concentration in the lab, kept on ice, and applied on site.

### 2.3. Mortality

Starting at placement, birds were monitored twice a day. For each mortality, the date, body weight and cause of death were recorded. This procedure continued throughout the study (up to d 42) to record mortality/treatment for each phase thus allowing for adjustment of performance parameters for daily mortality.

### 2.4. Lesion Scores

On d 8 of the study, two birds were selected based on average body weight of each pen (24/treatment), euthanized by cervical dislocation, and the small intestines were examined for NE lesions and scored based on a 0–4 scale system [23]. Each section of the small intestine, i.e., duodenum, jejunum and ileum, were scored separately by personnel blinded to the treatments. The lesion scoring criteria used were as follows:

0 = No gross lesions 1 = Thin-walled or friable 2 = Focal necrosis or ulceration 3 = Multifocal coalescing areas (large patches) of necrosis 4 = Severe extensive necrosis

### 2.5. Performance Parameters

Upon arrival (d 0), birds were randomly weighed in groups of 30 and assigned to each pen. Subsequently, birds were weighed by pen on d 8 (7 days after the coccidiosis challenge which was the peak mortality) and at the end of starter (d 14), grower 1 (d 21), grower 2 (d 28) and finisher (d 42) phases. Additionally, feed consumption was recorded on a per pen basis on days 8, 14, 21, 28, and 42. Finally, adjusted body weight gain, feed intake, and feed conversion ratios were calculated for each phase (d 0–8, 9–14, 15–21, 22–28, and 29–42) and also for the cumulative experimental period (d 0–42).

### 2.6. Gene Expression of Tight Junction Proteins, Cytokines, and Nutrient Transporters

On d 8, one bird/pen was selected, euthanized, and jejunum samples were excised to assess the gene expression of tight junction proteins. Jejunal tissue samples were homogenized by the TissueLyser II (Qiagen) and total RNA extracted using RNeasy Mini Kit (Qiagen GmbH, Hilden, Germany) according to the manufacturer’s instructions. Total RNA was quantified by spectrophotometry, and integrity evaluated by gel electrophoresis on 1.5% agarose gel in 0.5X TAE buffer. Two micrograms of total RNA were used to synthesize first-strand cDNA using the High Capacity cDNA Reverse Transcription Kit (Applied Biosystems, Carlsbad, CA, USA) according to the manufacturer’s recommendation. The abundance of tight junction proteins mRNA (claudin-1, claudin-3, zonula occluden-1, and zonula occluden-2), cytokines (IFN-γ, TNF-α, IL-1β, IL-10, IL-17, and IL-22), nutrient transporters (SGLT1 and PepT1), sIgA and AMP-activated protein kinase alpha 1 (AMPK-α1) were determined by quantitative real-time PCR (7500 Fast Real-Time PCR System, Applied Biosystems) using Fast SYBR^TM^ Green Master Mix (Applied Biosystems). Primer details are shown in Table 2. Each reaction was performed in a total volume of 20 μL in duplicate. Product specificity was confirmed by analysis of the melting curves produced by the 7500 software (version 2.0.3). Gene expression was analyzed using glyceraldehyde 3-phosphate dehydrogenase (GAPDH) as an endogenous control. Average gene expression relative to GAPDH for each sample was calculated using the 2^−ΔΔCt^ method [24]. The calibrator for each gene was the average ΔCt value from the negative control group [22].

### 2.7. Statistical Analysis

Statistical analysis for all data was performed using the ANOVA procedure of JMP software (2013) and significance between treatments (*p* ˂ 0.05) determined by the least significant difference (LSD) test. The statistical model for data analysis is outlined below:

Yij = μ + Ai + eij Yij = measured value for each observation (data) μ = grand mean Ai = treatment effect eij = experimental error

## 3. Results

### 3.1. Mortality

Only antibiotic growth promoter (AGP) reduced mortality significantly during d 0–8, and d 0–42 periods (Table 3). However, the probiotic was able to reduce d 0–42 mortality numerically compared to NC group. Moreover, probiotic and AGP treatments reduced mortality rate compared to NC between d 7–9 (peak mortality due to NE) and d 29–42. During the rest of the time periods, mortality was similar among the treatments.

### 3.2. Necrotic Enteritis Lesion Scores

Necrotic enteritis lesions were mostly prevalent in the duodenum and reduced through the distal parts of the small intestine. Probiotic Supplementation significantly reduced lesion scores in the duodenum and jejunum compared with NC, while virginiamycin (PC) only reduced lesion scores in the jejunum (Table 4).

### 3.3. Performance Parameters

Performance data are shown in Table 5. Average daily gain (ADG) was similar for all the treatments during the first two weeks, yet differences became evident by the third week. In the overall experimental period, the PC group had the highest ADG (77.69 g/bird), which was higher than NC (74.99 g/bird) but similar to the probiotic group. Feed intake data showed lower average daily feed intake (ADFI) for probiotic compared with NC and PC during d 22–28. Furthermore, for the overall experimental period (d 0–42) ADFI was significantly lower for the probiotic group compared to PC. During d 0–8, birds fed the diet containing probiotic had significantly lower feed conversion ratio (FCR) compared to PC but not NC. However, supplementation of probiotic and AGP improved FCR significantly compared to NC during d 29–42 and d 0–42.

### 3.4. Gene Expression of Tight Junction Proteins, Cytokines, and Nutrient Transporters

**Figure 1:**
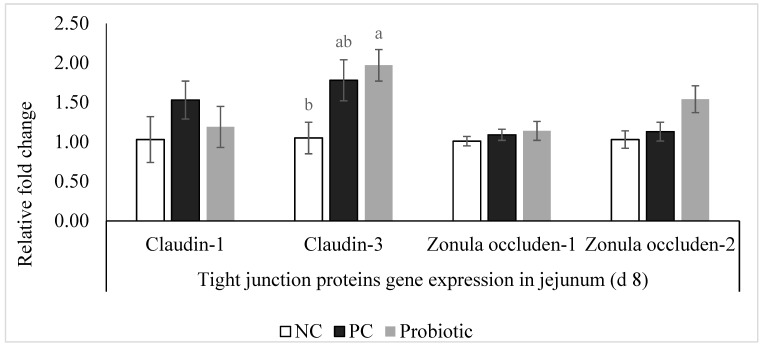
Relative gene expression of tight junction proteins in the jejunum of broiler chickens on d 8. Birds reared under a naturally occurring necrotic enteritis challenge model. Treatments include: negative control (NC): birds received a corn-soybean meal basal diet; positive control (PC): NC + virginiamycin (Stafac^®^20) at the level of 0.45 kg/ton from d 0-42; Probiotic: NC + PrimaLac at the level of 1.36 kg/ton in starter and grower 1 diets; 0.91 kg/ton in grower 2 and finisher diets. Values are represented as a *n*-fold difference relative to the calibrator (negative control, NC). Results are given as means (*n* = 12) for each treatment. Error bars indicate standard errors. For each gene, bars with different letters are significantly different (*p* < 0.05).

**Figure 2:**
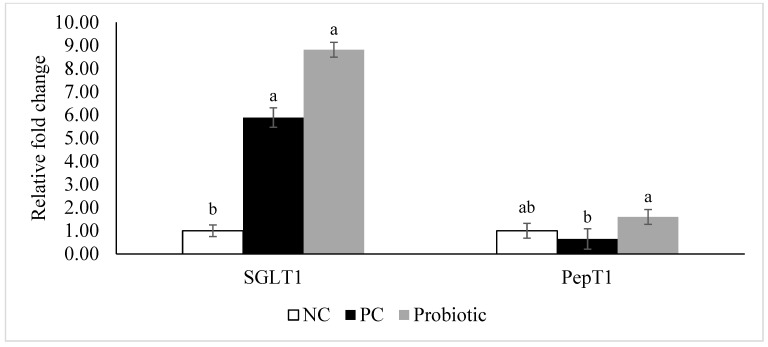
Relative gene expression of sodium-glucose co-transporter 1 (SGLT1) and peptide transporter 1 (PepT1) in the jejunum of broiler chickens on d 8. Birds reared under a naturally occurring necrotic enteritis challenge model. Treatments include: negative control (NC): birds received a corn-soybean meal basal diet; positive control (PC): NC + virginiamycin (Stafac^®^20) at the level of 0.45 kg/ton from d 0–42; Probiotic: NC + PrimaLac at the level of 1.36 kg/ton in starter and grower 1 diets; 0.91 kg/ton in grower 2 and finisher diets. Values are represented as a *n*-fold difference relative to the calibrator (negative control, NC). Results are given as means (*n* = 12) for each treatment. Error bars indicate standard errors. For each gene, bars with different letters are significantly different (*p* < 0.05).

**Figure 3:**
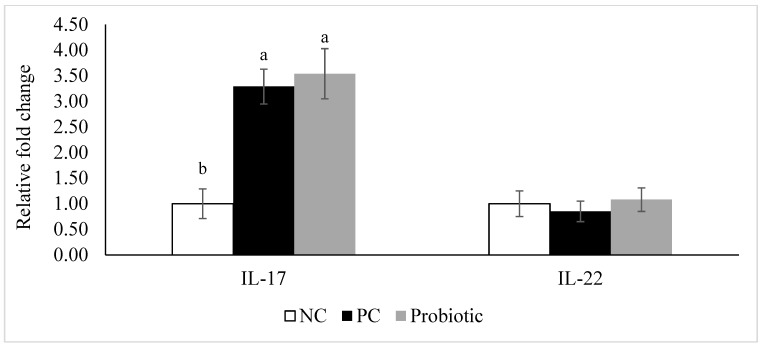
Relative gene expression of interleukin-17 (IL-17) and interleukin-22 (IL-22) in the jejunum of broiler chickens on d 8. Birds reared under a naturally occurring necrotic enteritis challenge model. Treatments include negative control (NC): birds received a corn-soybean meal basal diet; positive control (PC): NC + virginiamycin (Stafac^®^20) at the level of 0.45 kg/ton from d 0–42; Probiotic: NC + PrimaLac at the level of 1.36 kg/ton in starter and grower 1 diets; 0.91 kg/ton in grower 2 and finisher diets. Values are represented as a *n*-fold difference relative to the calibrator (negative control, NC). Results are given as means (*n* = 12) for each treatment. Error bars indicate standard errors. For each gene, bars with different letters are significantly different (*p* < 0.05).

**Figure 4:**
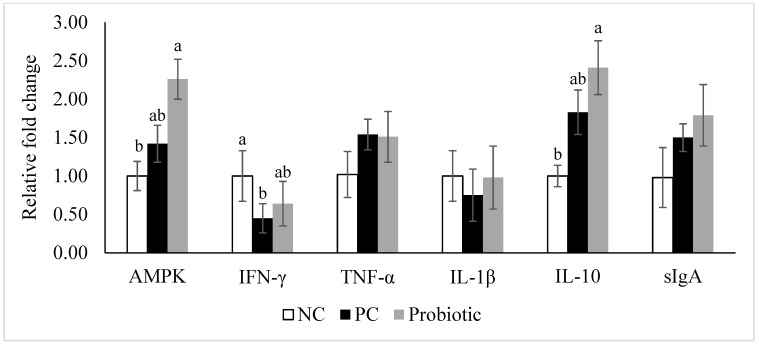
Relative gene expression of AMP-activated protein kinase alpha 1 (AMPK-α1), interferon-γ (IFN-γ), tumor necrosis factor-α (TNF-α), interleukin-1β (IL-1β), interleukin-10 (IL-10), and secretory immunoglobulin A (sIgA) in the jejunum of broiler chickens on d 8. Birds reared under a naturally occurring necrotic enteritis challenge model. Treatments include negative control (NC): birds received a corn-soybean meal basal diet; positive control (PC): NC + virginiamycin (Stafac^®^20) at the level of 0.45 kg/ton from d 0–42; Probiotic: NC + PrimaLac at the level of 1.36 kg/ton in starter and grower 1 diets; 0.91 kg/ton in grower 2 and finisher diets. Values are represented as a *n*-fold difference relative to the calibrator (negative control, NC). Results are given as means (*n* = 12) for each treatment. Error bars indicate standard errors. For each gene, bars with different letters are significantly different (*p* < 0.05).

Expression of claudin-3 was higher (*p* = 0.04) in the probiotic-supplemented birds compared to NC (Figure 1). Furthermore, despite similar levels for claudin-1 and zonula occluden-1, expression of zonula occluden-2 tended (*p* = 0.06) to be higher in the probiotic group than in the NC birds (Figure 1). Expression of IL-10, IL-17, AMPK-α1, and SGLT1 was significantly higher in the probiotic birds compared to the NC group, and probiotic supplemented birds had higher expression of PepT1 in the jejunum compared to PC (Figure 2 and Figure 3). For PC group, expression of IL-17 and SGLT1 was significantly higher than NC. There were no differences in gene expression of sIgA, TNF-α, IL-1β, and IL-22 among the treatments (Figure 4).

## 4. Discussion

In this study, we investigated the effect of a probiotic on performance and body composition of broiler chickens under a naturally occurring NE model. Furthermore, expression of tight junction proteins, cytokines, and nutrient transporters were evaluated in order to identify potential modes of action. The current findings revealed that the no-additive control group had lower (i.e., better) FCR compared to the AGP (PC) and probiotic groups, during d 0–8 and d 9–14, respectively. While this observation may seem intriguing, it may be due to the modulated immune response (higher expression of IL-10 and IL-17) and better gut health (lower lesion scores and higher expression of TJ proteins) in the probiotic and PC groups. These are energy demanding processes that divert nutrients from growth subsequently reducing mortality (lower during d 7–9 which is the peak NE mortality in this challenge model). However, birds fed diets supplemented with probiotic or virginiamycin (PC) had significantly lower (better) FCR in the grower 2 (d 22–28), finisher (d 29–42), and overall experimental period (d 0–42), indicating a compensatory growth of these birds following recovery from the disease challenge. Especially interesting was the better FCR for the probiotic group due to lower ADFI, while better FCR in the PC group was mostly the result of higher ADG. This suggests that probiotics could improve the efficiency of nutrient digestion and absorption which eventually leads to better FCR. This also was reflected by higher expression of nutrient transporters including SGLT1 and PepT1 in the jejunum of broilers fed the probiotic-supplemented diet. Nutrient transporters at the apical membrane of the small intestine are important in moving nutrients into the enterocytes. SGLT1 and PepT1 mediate absorption of carbohydrates (glucose and galactose) and di- and tri-peptides, respectively [27,28]. Therefore, these transporters are critical for maintaining the energy and amino acid supplies. SGLT1 is a co-transporter of glucose and sodium. Concurrent absorption of glucose and sodium establishes a gradient that facilitates the movement of sodium and water through the paracellular space [29]. This might be helpful in reducing diarrhea, which is a common symptom during enteric diseases, thus alleviating its negative impacts on the bird.

Gut health is very important when it comes to feed efficiency and FCR, and studies have shown the devastating effect of enteric diseases such as NE on FCR and profitability [5,8]. Birds in the probiotic-supplemented group exhibited the lowest lesion scores in the duodenum and jejunum on d 8 thus corroborating the hypothesis that supplementation of probiotic leads to better gut health. This was significantly lower than NC and could partially justify lower mortality in this group. Lower lesion scores are indicative of an intact, healthier, and more functional intestinal epithelium. Intact intestinal epithelium prevents entry of potential pathogens and leads to optimal health and performance of the bird as a result of proper nutrient absorption and utilization [13]. Tight junction proteins are the most important aspect of gut integrity and make up a barrier in the paracellular space. These proteins are subject to change and remodel in response to external stimuli in the gut lumen such as food/nutrients and commensal and pathogenic bacteria. Thus, these barriers are dynamic and subject to constant remodeling [30].

In the gastrointestinal tract, *C*. *perfringens* spores proliferate and produce enterotoxins. These enterotoxins (CPE) use claudin family proteins in the tight junction structure as binding sites/receptors and eventually lead to pore formation in host cells, and disruption of gut integrity as a result of this attachment [16,31]. An *in vitro* study reported that production of enterotoxins by *C*. *perfringens* type A increases in the presence of bile acids, which are secreted in the upper parts of the small intestine [32]. This might be a reason for higher lesion scores in the duodenum and jejunum compared with the ileum in the current study. 

Claudin family proteins have extracellular domains that are recognized by CPE as receptors, while other proteins in the tight junction structure such as zonula occludens, do not have extracellular domains and are only indirectly connected to extracellular space through attachment to claudins [16,33]. Expression of tight junction proteins mRNA showed higher levels of claudin-3 and zonula occluden-2 in the jejunum of birds fed the probiotic-supplemented diet compared to NC but not PC. This is an interesting finding showing that probiotics have the ability to modify tight junction protein structure while virginiamycin may have a different mode of action. Increase in claudin-3 and zonula occluden-2 expression might have resulted in better gut health and integrity, thus less lesions in the probiotic fed group. This might seem contradictory at first because CPE use claudin family proteins as binding sites and higher expression of claudin-3 could translate as more available binding sites for CPE. However, due to lower lesion scores in the jejunum and better FCR, we could conclude that modification of the tight junction protein complex as a result of probiotic supplementation led to the promotion of gut integrity and a healthier gut compared to NC. This is the first study in which we showed the possibility of tight junction modification in chickens through supplementation of probiotics under a field-like necrotic enteritis challenge.

Intestinal barrier function is regulated through intercellular and intracellular signaling systems, and the gut microbiota represent a key factor in orchestrating these signals and thus maintaining the gut barrier function [34]. Maintaining the barrier function of the gut is an energy dependent process. First, during infection and inflammation (e.g. NE), cell proliferation in the intestine (an energy consuming process) commonly occurs in order to replace damaged enterocytes [35]. Non-avian studies evidenced the role of IL-17 and IL-22, tissue-signaling cytokines that favor protection and regeneration of cells in barrier organs such as the skin, lung, and gastrointestinal tract [36]. In addition, IL-17A is important in inflammation and antimicrobial defense against pathogens (extracellular bacteria and fungi) at mucosal surfaces and regulates mucosal immune defenses [37,38]. In this study, higher expression of IL-17, but not IL-22, in the jejunum was observed in the PC and probiotic fed birds compared to NC on d 8. This might explain lower lesion scores in the PC and probiotic groups compared to NC due to the effect of IL-17 on epithelial cell regeneration, or lower damage due to the protective effects of IL-17 at mucosal surfaces. Park et al. [39] demonstrated that *C. perfringens* infection induced the expression of IL-17, which was significantly reduced following coinfection with *Eimeria maxima* and *C. perfringens* [39]. The latter seems to be the case in our experiment and supplementation of probiotics helped improve IL-17 expression compared to NC. In contrast, Wang et al. [40] reported that supplementation of a probiotic (*L*. *johnsonii* BS15) did not affect IL-17 expression in the duodenum during a mixed NE challenge model (*Eimeria* + CP). Another study reported numerically higher expression of IL-17 in antibiotic fed broiler chickens on d 7 post-challenge compared to a non-medicated control [41]. They concluded that CP infection induced an inflammatory response in the intestine of broiler chickens, and the mechanisms of inflammation are probably mediated via Th2 and Th17 cells. Discrepancies in the results could be attributed to several factors including the use of different challenge models, various probiotics, and potentially different sampling sites.

Further, during inflammation and infection, mitochondrial function is disrupted (increased reactive oxygen species production), which negatively affects paracellular permeability [42]. Increasing evidence indicates that AMPK, the master regulator of energy metabolism in the cell, promotes the formation of tight junctions in epithelial cells and is critical in the restructuring of tight junctions. Additionally, AMPK activation enhances paracellular junctions and nutrient transporters and suppresses inflammation in the intestine, indicating an essential role of AMPK in intestinal health [43,44]. Lack of AMPK-α1 in mice leads to the less compact ultrastructure of tight junctions and thus higher intestinal permeability [21]. Pro-inflammatory cytokines are primarily produced by lamina propria macrophages upon confrontation with bacteria and could trigger the activation of T cells and neutrophils [45]. IL-1 and TNF-α represent the archetypal pro-inflammatory cytokines that are rapidly released upon tissue injury or infection [46]. Pro-inflammatory cytokines, including IFN-γ, could affect tight junction structure through suppressing AMPK expression and are thus etiological factors in intestinal barrier dysfunction [43,44]. In conjunction with Wang et al. [40], who reported similar expression of LITAF in the ileum of non-medicated vs. probiotic group, TNF-α expression in the jejunum was not affected by antibiotic or probiotic supplementation in our study. In addition, there was no significant difference between treatments with regard to IL-1β in our experiment, while IFN-γ expression was lower in PC compared to NC group. Similarly, others [41] reported that expression of IFN-γ was significantly lower in challenged birds fed antibiotic (BMD) compared to challenged control birds.

Expression of IL-10 was significantly higher in the probiotic supplemented group compared to NC, which is accordance with lower lesion scores in this group. During infection with protozoa and bacteria, IL-10 acts as an immune regulator and ameliorates excessive Th1 and CD8+ T cell responses [47,48]. Th1 responses are necessary for dealing with *Eimeria* infections [49] and help to maximize clearance of pathogens, which may cause tissue damage [47]. IL-10 was also shown to be involved in the restoration of the epithelial barrier and a lack of or reduced production of IL-10 by macrophages compromises the recovery of the small intestine epithelial barrier in mice [50]. Thus, regulatory effects of IL-10 might have contributed to the effectiveness of the probiotic.

Based on the presented findings, it could be concluded that under a naturally occurring necrotic enteritis challenge model, supplementation of probiotic to the diet of broiler chickens significantly reduced intestinal lesion scores on d 8 and improved FCR during the overall growth period. Moreover, dietary addition of a probiotic improved intestinal barrier function by regulating the tight junction proteins gene expression and mucosal immune responses.

## Figures and Tables

**Table 1:** Composition of basal diets (as fed basis, %) ^1^.

	Feeding Phase (Days)		
Ingredients (%)	Starter (0–14)	Grower 1 + 2 (15–28)	Finisher (29–42)
Corn (7.81% crude protein)	59.53	64.12	65.70
Soybean meal (48% crude protein)	33.5	28.80	26.86
Soybean Oil (9000 kcal/kg)	2.18	2.60	3.50
Dicalcium Phosphate (18.5% Phosphorus, 22% Calcium)	2.05	1.92	1.70
Calcium Carbonate (37% Calcium)	1.11	1.00	0.90
Sodium Chloride	0.37	0.37	0.35
DL-Methionine (990 g/kg) ^2^	0.38	0.34	0.29
L-Lysine Hydrochloride (788 g L-Lysine/kg) ^3^	0.37	0.35	0.24
L-Threonine (985 g/kg) ^4^	0.15	0.14	0.10
Vitamin/Trace Mineral Premix ^5^	0.36	0.36	0.36
**Calculated Analysis (% unless specified)**			
ME (kcal/kg)	3007	3087	3168
Crude protein	21.81	19.90	18.94
Total phosphorus	0.76	0.71	0.66
Available phosphorus	0.45	0.42	0.38
Calcium	0.90	0.84	0.76
Chlorine	0.33	0.33	0.29
Sodium	0.16	0.16	0.15
Potassium	0.85	0.77	0.73
Methionine	0.67	0.61	0.55
Methionine + Cysteine	0.98	0.89	0.82
Lysine	1.32	1.19	1.05
Threonine	0.86	0.78	0.71
Linoleic acid	1.44	1.52	1.55
Dietary cation-anion balance (mEq)	194	174	170

^1^ The supplemental antibiotic growth promoter (0.45 kg/ton of Stafac^®^20), and PrimaLac (1.36 kg/ton in starter and grower 1 diets; 0.91 kg/ton in grower 2 and finisher diets) were added on top to the basal diet to provide the three experimental diets including the control (basal) diet in every feeding period. ^2^ Rhodimet® NP99, ADISSEO, GA, USA. ^3^ L-Lysine HCl, Ajinomoto Heartland, Inc. Eddyville, IA, USA. ^4^ Fenchem Ingredient Technology, Nanjing, China. ^5^ Vitamins supplied per kg diet: retinol 3.33 mg, cholecalciferol 0.1 mg, α-tocopherol acetate 23.4 mg, vitamin K3 1.2 mg, vitamin B1 1.6 mg, vitamin B2 9.5 mg, niacin 40 mg, pantothenic acid 9.5 mg, vitamin B6 2 mg, folic acid 1 mg, vitamin B12 0.016 mg, biotin 0.05 mg, choline 556 mg. Minerals supplied per kg diet: Mn 144 mg, Fe 72 mg, Zn 144 mg, Cu 16.2 mg, I 2.1 mg, Se 0.22 mg.

**Table 2:** Sequences of primer pairs used for amplification of target and reference genes. For each gene, the primer sequences for forward (F) and reverse (R) are listed (5′-3′), the amplicon size (bp) and the NCBI Accession number (Acc) used for the primer design.

Gene	Primer Sequence	Size	Acc (Reference)
Claudin-1	GTGTTCAGAGGCATCAGGTATC GTCAGGTCAAACAGAGGTACAA	107	NM_001013611.2
Claudin-3	CCCGTCCCGTTGTTGTTTTG CCCCTTCAACCTTCCCGAAA	126	NM_204202.1 [25]
Zonula occluden-1	GGAGTACGAGCAGTCAACATAC GAGGCGCACGATCTTCATAA	101	XM_413773
Zonula occluden-2	GCGTCCCATCCTGAGAAATAC CTTGTTCACTCCCTTCCTCTTC	89	NM_204918
Interferon-γ	GCTCCCGATGAACGACTTGA TGTAAGATGCTGAAGAGTTCATTCG	63	NM_205149.1
TNF-α ^1^	CCCATCCCTGGTCCGTAAC ATACGAAGTAAAGGCCGTCCC	77	MF000729.1
sIgA ^2^	GTCACCGTCACCTGGACTACA ACCGATGGTCTCCTTCACATC	192	S40610
Interleukin-1β	CCCGCCTTCCGCTACA CACGAAGCACTTCTGGTTGATG	66	XM_015297469.1
Interleukin-10	CGCTGTCACCGCTTCTTCA CGTCTCCTTGATCTGCTTGATG	63	NM_001004414.2
Interleukin-17A	AGCTGGACCACAGCGTCAAC GGCGGAGGACGAGGATCT	57	NM_204460.1
AMPK-α1 ^3^	ATCTGTCTCGCCCTCATCCT CCACTTCGCTCTTCTTACACCTT	125	NM_001039603
SGLT1 ^4^	GCCATGGCCAGGGCTTA CAATAACCTGATCTGTGCACCAGTA	71	NM_0,012,93240.1 [26]
PepT1 ^5^	CCCCTGAGGAGGATCACTGTT CAAAAGAGCAGCAGCAACGA	66	NM_204,365.1 [26]
GAPDH ^6^	CCTAGGATACACAGAGGACCAGGTT GGTGGAGGAATGGCTGTCA	64	NM_204305

^1^ Tumor necrosis factor-α. ^2^ Secretory immunoglobulin-A. ^3^ AMP-activated protein kinase. ^4^ Sodium-glucose co-transporter 1. ^5^ Peptide transporter 1. ^6^ Glyceraldehyde 3-phosphate dehydrogenase.

**Table 3:** Effect of antibiotic growth promoter or probiotic supplementation in the diet on mortality of broiler chickens under naturally occurring necrotic enteritis challenge.

Treatments ^1^	Time Period (day)						
	0–8	7–9	9–14	15–21	22–28	29–42	0–42
NC	5.28 ^a^	4.44 ^a^	2.23	0.00	1.64	3.60 ^a^	11.39 ^a^
PC	0.28 ^b^	0.55 ^b^	1.50	0.31	0.00	0.00 ^b^	1.94 ^b^
Probiotic	4.72 ^a^	1.51 ^b^	0.95	0.64	0.73	0.42 ^b^	6.94 ^ab^
*SEM* ^2^	*1.39*	*0.94*	*0.61*	*0.31*	*0.53*	*0.73*	*1.79*
*p-value*	*0.030*	*0.018*	*0.347*	*0.347*	*0.106*	*0.002*	*0.003*

^a,b^ In each column, means with different letters are significantly different (*p* < 0.05). ^1^ Treatments include: negative control (NC): birds received a corn-soybean meal basal diet; positive control (PC): NC + virginiamycin (Stafac^®^20) at the level of 0.45 kg/ton from d 0–42; Probiotic: NC + PrimaLac at the level of 1.36 kg/ton in starter and grower 1 diets; 0.91 kg/ton in grower 2 and finisher diets. ^2^ SEM: Standard error of means.

**Table 4:** Effect of antibiotic growth promoter or probiotic supplementation in the diet on intestinal lesion scores of broiler chickens under naturally occurring necrotic enteritis challenge.^1^

Treatments ^2^	Small Intestine Section		
	Duodenum	Jejunum	Ileum
NC	2.25 ^a^	1.32 ^a^	0.32
PC	1.77 ^ab^	0.85 ^b^	0.12
Probiotic	1.41 ^b^	0.86 ^b^	0.34
*SEM* ^3^	*0.17*	*0.14*	*0.16*
*p-value*	*0.006*	*0.040*	*0.582*

^a,b^ In each column, means with different letters are significantly different (*p* < 0.05). ^1^ Data represent the mean value of 12 replicate pens of 2 birds/pen. ^2^ Treatments include: negative control (NC): birds received a corn-soybean meal basal diet; positive control (PC): NC + virginiamycin (Stafac^®^20) at the level of 0.45 kg/ton from d 0–42; Probiotic: NC + PrimaLac at the level of 1.36 kg/ton in starter and grower 1 diets; 0.91 kg/ton in grower 2 and finisher diets. ^3^ SEM: Standard error of means.

**Table 5:** Effect of antibiotic growth promoter or probiotic supplementation in the diet on broiler performance under a naturally occurring necrotic enteritis model.^1^

	Dietary Treatments ^2^				
Item ^3^	NC	PC	Probiotic	*SEM* ^4^	*p*-Value
d 0–8					
ADFI, g	23.28	23.50	23.31	*0.24*	*0.784*
ADG, g	17.1	16.59	17.29	*0.25*	*0.137*
FCR, g/g	1.36 ^ab^	1.41 ^a^	1.35 ^b^	*0.02*	*0.053*
d 9-14					
ADFI, g	57.21	56.09	56.84	*0.64*	*0.474*
ADG, g	41.97	40.38	40.59	*0.59*	*0.134*
FCR	1.36 ^b^	1.39 ^ab^	1.40 ^a^	*0.01*	*0.043*
d 15–21					
ADFI, g	106.10	105.41	104.55	*1.34*	*0.708*
ADG, g	71.15	70.65	68.84	*1.42*	*0.468*
FCR	1.49	1.49	1.52	*0.02*	*0.414*
d 22–28					
ADFI, g	161.89 ^a^	159.72 ^a^	154.75 ^b^	*1.75*	*0.018*
ADG, g	103.07	105.47	103.78	*1.62*	*0.567*
FCR	1.57 ^a^	1.51 ^b^	1.49 ^b^	*0.01*	*˂0.001*
d 29–42					
ADFI, g	202.19	203.59	199.85	*2.17*	*4.74*
ADG, g	113.00 ^b^	119.53 ^a^	116.00 ^ab^	*1.55*	*0.020*
FCR	1.79 ^a^	1.70 ^b^	1.72 ^b^	*0.01*	*0.001*
d 0–42					
ADFI, g	119.45 ^ab^	120.62 ^a^	116.80 ^b^	*1.11*	*0.050*
ADG, g	74.99 ^b^	77.69 ^a^	75.72 ^ab^	*0.85*	*0.048*
FCR	1.59 ^a^	1.55 ^b^	1.54 ^b^	*0.01*	*˂0.001*

^a,b^ Within each row, means with different letters are significantly different (*p* < 0.05). ^1^ Data represent the mean value of 12 replicate pens of 30 birds. ^2^ Treatments include: negative control (NC): birds received a corn-soybean meal basal diet; positive control (PC): NC + virginiamycin (Stafac^®^20) at the level of 0.45 kg/ton from d 0-42; Probiotic: NC + PrimaLac at the level of 1.36 kg/ton in starter and grower 1 diets; 0.91 kg/ton in grower 2 and finisher diets. ^3^ ADFI: Average daily feed intake, ADG: average daily gain, FCR: feed conversion ratio. ^4^ SEM: Standard error of means.
